# Improved 18S and 28S rDNA primer sets for NGS-based parasite detection

**DOI:** 10.1038/s41598-019-52422-z

**Published:** 2019-10-31

**Authors:** Asuka Kounosu, Kazunori Murase, Akemi Yoshida, Haruhiko Maruyama, Taisei Kikuchi

**Affiliations:** Division of Parasitology, Faculty of Medicine, University of Miyazaki, Miyazaki, 889-1692 Japan

**Keywords:** Metagenomics, Parasitology

## Abstract

The development and application of next-generation sequencing (NGS) have enabled comprehensive analyses of the microbial community through extensive parallel sequencing. Current analyses of the eukaryotic microbial community are primarily based on polymerase chain reaction amplification of 18S rRNA gene (rDNA) fragments. We found that widely-used 18S rDNA primers can amplify numerous stretches of the bacterial 16S rRNA gene, preventing the high-throughput detection of rare eukaryotic species, particularly in bacteria-rich samples such as faecal material. In this study, we employed *in silico* and NGS-based analyses of faecal samples to evaluated the existing primers targeting eukaryotic 18S and 28S rDNA in terms of avoiding bacterial read contamination and improving taxonomic coverage for eukaryotes, with a particular emphasis on parasite taxa. Our findings revealed that newly selected primer sets could achieve these objectives, representing an alternative strategy for NGS.

## Introduction

Next-generation sequencing (NGS) using the 16S rRNA gene (16S rDNA) has been widely used to examine bacterial diversity^1,2^. In our previous study, we applied NGS using the 18S rRNA gene (18S rDNA) to analyse eukaryotic parasite diversity^3,4^. Compared with conventional methods which rely on host dissections and/or microscopic observations, NGS-based methods are easy and sufficiently sensitive for high-throughput analyses^3,4^.

18S rDNA has been widely used for the identification and diversity analyses of eukaryotes because it is well conserved among species and it contains variable regions^5,6^. Within 18S rDNA, hypervariable regions 4 (V4) and 9 (V9) are currently the popular options for NGS-based analyses^5–8^. The Earth Microbiome Project (EMP), which aims to construct a global catalogue of the uncultured microbial diversity on the Earth^9^, recommends the use of primers that amplify a short fragment (approximately 150 bp) containing the V9 region of 18S rDNA for eukaryote analyses. As with those studies using PCR, primer selection is a critical factor for successful NGS-based analyses because non-universal primers are subject to taxonomic biases. Along with the elongation of the read length by the Illumina sequencer, some recent studies seeking to develop improved primer sets which amplify longer fragments have compared 18S rDNA among all eukaryotes^5,6^ or specific taxa^8^ via *in silico* sequence analysis and identified conserved regions best suited for amplifying the hypervariable regions. However, although those primer sets were designed to amplify eukaryotic 18S rDNA fragments, several bacterial 16S rDNA fragments were also amplified^10–12^, indicating their poor specificity. Specifically, for bacteria-rich samples, such as faecal material, bacterial read contamination represents a critical drawback, preventing the detection of rare eukaryotic species. In addition, refined classification of the detected reads to the genus or species level is often difficult using 18S rDNA primers because the amplicon sequence does not represent sufficient sequence diversity to distinguish closely related genera or species. Other genomic regions, such as the large subunit (LSU) of rDNA, which varies from 25S to 28S in size depending on the species (in this article we use “28S rDNA” to refer eukaryotic LSU), or the ITS regions of rDNA, which show higher diversity than 18S rDNA, represent alternative targets for PCR amplification^13,14^.

In this study, we sought to identity primer sets that provide high taxonomic resolution and less bacterial read contamination to investigate eukaryotic microbial diversity with a particular emphasis on parasitic taxa. Primer screening was performed using 18S and 28S rDNA via *in silico* sequence analyses, and selected primers were further evaluated for sensitivity, specificity, taxonomic discrimination capacity, amplification efficiency and reproducibility via quantitative PCR (qPCR) and NGS analyses of faecal samples obtained from parasite-infected animals.

## Results

### *In silico* screening

For NGS-based analyses of eukaryote diversity, previous studies have mostly used 1391F/EukBr, as recommended by EMP^10,15–17^, or 563F/1132R^18–22^, which targets the V9 or V4-5 regions of 18S rDNA, respectively. In this article, these two primer sets are referred to as ‘conventional primer sets’ and used as comparators. To identify primer pairs that can efficiently detect a wide variety of parasites while avoiding bacterial DNA amplification for use in NGS-based parasite detection, we screened all possible 18S and 28S rDNA primers. Some previous studies have extensively tested 18S rDNA primers *in silico* to design universal eukaryotic primers to be used as standards for NGS-based analyses of eukaryote diversity^5,6,13^. Therefore, we used these recommended 18S rDNA primer sets to re-evaluate for detection of parasitic taxa groups (Table 1; Supplementary Table S1). For 28S rDNA, we retrieved the possible universal primers (n = 52) (Supplementary Table S1) from previous reports^13,23,24^ and screened them based on their melting temperatures (Tm) and amplicon sizes (Materials and Methods), yielding 13 primer pairs (Table 2).

**Table 1:** List of primer sets targeting the 18S rRNA gene and their coverage in 14 taxonomic groups.

Taxonomy^a^	Representative species	EMP(1391F/EukBr)^b,c^		563F/1132R^c^		574F/952R^c^		574*F/952R^c^		616*F/1132R^c^		1183F/1631R^c^	
		V9		V4–V5		V4–V5		V4–V5		V4–V5		V7–V8	
		145 bp		569 bp		378 bp		378 bp		516 bp		449 bp	
Nematoda (2169)	Roundworm, Filaria	88.2	(551/625)	95.9	(2072/2160)	42.0	(909/2163)	93.2	(2015/2163)	96.0	(2080/2166)	88.0	(1824/2072)
Platyhelminthes (1963)	Tapeworm, Fluke	81.8	(306/374)	88.4	(1713/1937)	89.2	(1734/1945)	88.9	(1729/1945)	88.7	(1727/1947)	90.1	(1713/1901)
Acanthocephala (63)	Spiny-headed worm	100.0	(26/26)	95.2	(60/63)	0.0	(0/63)	0.0	(0/63)	93.7	(59/63)	0.0	(0/62)
Coccidia (671)	Coccidium	96.0	(168/175)	97.9	(656/670)	96.7	(649/671)	96.7	(649/671)	97.2	(652/671)	90.8	(444/489)
Cryptosporida (55)	Cryptosporidium	97.3	(36/37)	94.5	(52/55)	96.4	(53/55)	96.4	(53/55)	92.7	(51/55)	100.0	(53/53)
Haemosporidia (149)	Plasmodium	87.5	(63/72)	0.0	(0/148)	98.0	(145/148)	98.0	(145/148)	0.0	(0/149)	0.0	(0/95)
Fornicata (21)	Giardia	100.0	(7/7)	100.0	(21/21)	23.8	(5/21)	33.3	(7/21)	71.4	(15/21)	30.0	(6/20)
Discicristata (909)	Trypanosoma, Leishmania	92.2	(306/332)	93.0	(806/867)	68.0	(608/894)	69.9	(663/949)	71.7	(640/893)	86.2	(698/810)
Parabasalia (303)	Trichomonas	77.0	(47/61)	98.0	(297/303)	0.0	(0/303)	44.2	(134/303)	19.8	(60/303)	0.0	(0/299)
Entamoebida (4)	Entamoeba	100.0	(3/3)	100.0	(4/4)	100.0	(4/4)	100.0	(4/4)	100.0	(4/4)	0.0	(0/3)
Longamoebia (158)	Acanthamoeba	73.6	(39/53)	87.4	(125/143)	89.0	(130/146)	90.4	(132/146)	88.0	(132/150)	89.5	(119/133)
Fungi (15101)		56.4	(3076/5458)	96.6	(14501/15006)	45.3	(1434/3164)	95.6	(14412/15071)	93.7	(14156/15100)	91.9	(13132/14290)
Bacteria (575268)		37.6	(34016/90407)	89.9	(516999/58262)	<0.1	(12/575261)	<0.1	(14/575247)	<0.1	(252/575265)	<0.1	(1/558088)
Euteleostomi (1091)	Mammal^d^	55.0^h,m^	(382/694)	75.5^h,m^	(816/1081)	72.5^h,m^	(784/1081)	61.6^h,m^	(788/1081)	75.0^h,m^	(815/1087)	62.5^h,m^	(599/958)

Primer sets were tested for matches to sequences in the SILVA database (v.132) using TestPrime under the following parameters: maximum number of mismatches of four bases and length of 0-mismatch at the 3′ end of three bases). TestPrime computes coverages for each taxonomic group by running *in silico* PCR on the SILVA database via sorting database sequences into “match”, “mismatch” and “nodata (sequences not covering the primer match position)”. The frequencies of “match” sequences among “match” and “mismatch” sequences are shown as percentages with the sequence numbers in parentheses.

^a^Numbers in parentheses show the numbers of sequences available in the SILVA database. Please note these numbers are not always the denominators because of the presence of “nodata” sequences.

^b^Only 17 bases from 3′ was used for the primer EukBr because many sequences in the SILVA database lacks the corresponding 5′ region.

^c^Target variable regions and amplicon sizes based on the *S. cerevisiae* rRNA gene (NC_001144) are shown below the primer names.

^d^Primer match to humans and mice is indicated by superscripts h and m on the values, respectively.

**Table 2:** List of primer sets targeting the 28S rRNA gene and their coverages in 14 taxonomic groups.

Taxonomy	Representative species	DM568F/RM2R^b^		DM568F/GA13R^b^		DM568F/RM3R^b^		RM2F/RM3R^b^		RM2F/GA15R^b^		GA12F/RM4R^b^	
		D3–D4		D3–D4		D3–D5		D4–D5		D4–D5		D4–D6	
		284 bp		327 bp		500 bp		236 bp		369 bp		507 bp	
Nematoda (659)	Roundworm, Filaria	62.1	(339/546)	42.5	(232/546)	62.1	(339/546)	94.7	(551/582)	54.8	(319/582)	92.9	(546/588)
Platyhelminthes (571)	Tapeworm, Fluke	88.2	(439/498)	84.9	(423/498)	91.4	(455/498)	95.2	(496/521)	23.2	(121/521)	97.3	(510/524)
Acanthocephala (56)	Spiny-headed worm	94.6	(53/56)	69.6	(39/56)	94.6	(53/56)	94.6	(53/56)	28.6	(16/56)	100.0	(56/56)
Coccidia (76)	Coccidium	90.4	(66/73)	91.8	(67/73)	90.4	(66/73)	91.9	(68/74)	83.8	(62/74)	93.2	(69/74)
Cryptosporida (1)	Cryptosporidium	100.0	(1/1)	100.0	(1/1)	100.0	(1/1)	100.0	(1/1)	100.0	(1/1)	100.0	(1/1)
Haemosporidia (115)	Plasmodium	100.0	(89/89)	0.0	(0/89)	95.5	(85/89)	94.9	(93/98)	87.8	(86/98)	0.0	(0/101)
Fornicata (5)	Giardia	20.0	(1/4)	0.0	(0/5)	0.0	(0/5)	0.0	(0/5)	20.0	(1/4)	40.0	(2/5)
Discicristata (35)	Trypanosoma, Leishmania	67.7	(21/31)	61.3	(19/31)	41.9	(13/31)	40.4	(13/32)	75.0	(24/32)	100.0	(33/33)
Parabasalia (20)	Trichomonas	100.0	(17/17)	94.1	(16/17)	0.0	(0/17)	0.0	(0/18)	61.1	(11/18)	5.6	(1/18)
Entamoebida (9)	Entamoeba	100.0	(5/5)	0.0	(0/5)	0.0	(0/5)	0.0	(0/8)	0.0	(0/8)	0.0	(0/8)
Longamoebia (1)	Acanthamoeba	100.0	(1/1)	100.0	(2/2)	100.0	(1/1)	100.0	(2/2)	100.0	(1/1)	100.0	(1/1)
Fungi (3671)		81.8	(2876/3515)	93.2	(3277/3515)	94.1	(3308/3515)	93.6	(3347/3576)	85.1	(3044/3576)	95.5	(3416/3578)
Bacteria (124805)		<0.1	(12/122765)	<0.1	(5/122772)	<0.1	(9/122777)	<0.1	(9/123872)	<0.1	(22/123872)	<0.1	(5/124043)
Euteleostomi (445)	Mammal^c^	74.7^h,m^	(236/316)	75.3^h,m^	(238/316)	77.2^h,m^	(244/316)	79.5^h,m^	(303/381)	75.3^h,m^	(287/381)	82.7^h,m^	(324/392)
**RM3F/RM4R** ^**b**^		**GA14F/RM4R** ^**b**^		**GA18F/RM7R** ^**b**^		**GA18F/RM8R** ^**b**^		**GA20F/RM7R** ^**b**^		**GA20F/RM8R** ^**b**^		**GA20F/RM9R** ^**b**^	
**D6**		**D6**		**D8**		**D8**		**D8**		**D8-D9**		**D8-D9**	
**333 bp**		**202 bp**		**424 bp**		**572 bp**		**348 bp**		**406 bp**		**505 bp**	
92.5	(564/610)	95.5	(592/620)	95.9	(626/653)	95.8	(619/646)	97.7	(638/653)	97.8	(632/646)	98.0	(632/645)
97.8	(523/535)	96.7	(526/544)	86.3	(465/539)	89.0	(471/529)	91.7	(493/539)	94.5	(500/529)	96.6	(510/528)
100.0	(56/56)	35.7	(20/56)	96.4	(54/56)	100.0	(56/56)	96.4	(54/56)	100.0	(56/56)	100.0	(56/56)
91.9	(68/74)	91.9	(68/74)	97.1	(68/70)	95.5	(64/67)	98.6	(69/70)	97.0	(65/67)	98.5	(65/66)
100.0	(1/1)	100.0	(1/1)	100.0	(1/1)	100.0	(1/1)	100.0	(1/1)	100.0	(1/1)	100.0	(1/1)
95.2	(99/104)	98.1	(104/106)	61.1	(69/113)	61.6	(69/112)	92.0	(104/113)	97.3	(109/112)	97.3	(109/112)
20.0	(1/5)	60.0	(3/5)	0.0	(0/5)	0.0	(0/5)	100.0	(5/5)	100.0	(5/5)	100.0	(5/5)
57.1	(20/35)	100.0	(35/35)	48.6	(17/35)	45.7	(16/35)	62.9	(22/35)	60.0	(21/35)	62.9	(22/35)
0.0	(0/20)	0.0	(0/20)	0.0	(0/20)	36.8	(7/19)	0.0	(0/20)	94.7	(18/19)	94.7	(18/19)
100.0	(9/9)	100.0	(9/9)	0.0	(0/9)	0.0	(0/9)	0.0	(0/9)	0.0	(0/9)	0.0	(0/9)
100.0	(1/1)	100.0	(1/1)	100.0	(2/2)	100.0	(1/1)	100.0	(1/1)	100.0	(1/1)	100.0	(1/1)
94.7	(3425/3617)	95.5	(3469/3631)	90.7	(2943/3244)	88.6	(2839/3205)	95.2	(3087/3244)	92.5	(2966/3205)	93.8	(3006/3205)
<0.1	(5/124626)	<0.1	(6/124788)	<0.1	(13/124775)	<0.1	(10/124065)	<0.1	(13/124775)	<0.1	(10/124065)	<0.1	(23/124041)
83.3^h,m^	(340/408)	82.3^h,m^	(340/413)	74.2^h,m^	(316/426)	75.6^h,m^	(304/402)	72.3^h,m^	(308/426)	73.6^h,m^	(296/402)	75.3^h,m^	(302/401)

Primer sets were tested for matches to sequences in the SILVA database (v.132) using TestPrime under the following parameters: maximum number of mismatches of four bases and length of 0-mismatch at the 3′ end of three bases). TestPrime computes coverages for each taxonomic group by running *in silico* PCR on the SILVA database via sorting database sequences into “match”, “mismatch” and “nodata (sequences not covering the primer match position)”. The frequencies of “match” sequences among “match” and “mismatch” sequences are shown as percentages with the sequence numbers in parentheses.

^a^Numbers in parentheses show the numbers of sequences available in the SILVA database. Please note these numbers are not always the denominators because of the presence of “nodata” sequences.

^b^Target variable regions and amplicon sizes based on the *S. cerevisiae* rRNA gene (NC_001144) are shown below the primer names.

^c^Primer match to humans and mice is indicated by superscripts h and m on the values, respectively.

We then evaluated those primer pairs based on their sequence identity with eukaryotic parasites, fungi and bacteria using the SILVA non-redundant sequence dataset (Tables 1 and 2). The EMP primer set (1391F/EukBr) can be used to detect a wide variety of parasitic taxa, exhibiting 43.4–66.7% coverage in majority of the tested taxa, excluding Nematoda, Platyhelminthes, Longamoebia and Fungi. The other conventional primer set 563F/1132R exhibited higher coverage than the EMP primer set (≥87.4% excluding Haemosporodia). However, these two primer sets showed similarity with bacterial 16S rDNA sequences (13.0% and 89.9%, respectively). Although the other 18S primer sets demonstrated lower taxonomic coverage for eukaryotes than 563F/1132R, they appeared to amplify less bacterial rDNA. For instance, 574F/952R showed low coverage for Nematoda, Fornicata and Parabasalia. Moreover, 574F/952R, 574*F/952R and 1183F/1631R showed low coverage for Acanthocephala, while 1183F/1631R showed low coverage for Parabasalia, Haemosporidia and Entamoebida, despite their low similarities to bacterial sequences (coverage <0.1%). These tendencies were also observed when we used strict or mild parameters for taxonomic coverage evaluations (Tables S2 and S3). Based on these results, 616*F/1132R and 1183F/1631R were selected for further evaluation as the best primer sets for the 18S V4-5 and V7-8 regions, respectively.

Data for the 28S rDNA primers are summarised in Tables 2, S2 and S3. All 28S primer sets showed low similarity with bacterial sequences. The taxonomic coverage for some eukaryotic parasites was variable, especially for some protozoan groups including Haemosporidia, Fornicata, Parabasalia and Entamoebida. On the other hand, the coverage for the other parasitic taxa were not very different, although the primer sets designed for the D3–D5 regions showed low coverage for Nematoda, Platyhelminthes and Discicristata. Among the five primer sets designed based on the D8–D9 regions, GA20F/RM8R, RM7F/RM9R and GA20F/RM9R exhibited wide taxonomic coverage except for Entamoebida. Based on these results, we selected seven 28S primer sets, namely DM568F/RM2R, RM2F/RM3R, RM3F/RM4R, GA12F/RM4R, GA20F/RM7R, GA20F/RM8R and GA20F/RM9R, for further evaluation.

All the 18S and 28S primers tested showed high coverages (>60%) for Euteleostomi which includes their possible hosts. In particular, human and mouse DNA are likely to be amplified by all primers tested (Tables 1 and 2).

### qPCR

To confirm whether the selected primer sets could efficiently amplify eukaryotic rDNA without dimer or hairpin structure generation, we performed qPCR using *C. elegans* DNA as representative eukaryote DNA because all selected primers displayed 100% sequence similarity with *C. elegans* rRNA. 18S rDNA from 0.1 ng of *C. elegans* genomic DNA (final concentration, 0.01 ng/µl), which corresponds to ~200,000 copies of rRNA, was amplified at ~21 cycles (mean Ct ± SD = 21.28 ± 0.71) using the EMP primer set (1391F/EukBr), corresponding to the amplification efficiency of 80–87%. 1183F/1631R and 563F/1132R exhibited similar PCR efficiencies, whereas 616*F/1132R showed lower efficiency (Supplementary Fig. S1A). To assess the avoidance of bacterial DNA amplification, we used a bacterial DNA mixture. Amplification from 0.1 ng of bacterial DNA (final concentration of 0.01 ng/µl), which corresponds to ~20000 copies of rRNA, required ~27 cycles (mean Ct ± SD = 27.10 ± 0.81) using the EMP primer set. Ct difference between eukaryotic and bacterial DNA was the largest for 1183F/1631R, followed by 616*F/1132R, whereas this difference was the smallest for the EMP primer set. The results for 28S rDNA primer sets are shown in Supplementary Fig. S1B. Amplification efficiencies of the 28S primer sets for *C. elegans* DNA exceeded 70% except for that of RM3F/RM4R, which required 12–16 additional cycles for amplification. All 28S primers demonstrated lower sensitivity to bacterial DNA and required more than 10 additional PCR cycles compared with the EMP primer set.

The detection limits of *C. elegans* DNA using the primer sets were 0.2–2 pg, corresponding to 1–10 *C. elegans* cells. Products were detected for no-template negative controls at approximately 30 cycles using RM2F/RM3R compared with approximately 35 cycles using the EMP primer sets. No non-specific amplification was detected with 40 cycles using the other primer sets.

Based on these results, we selected one primer set for each variable region of 28S rDNA, namely DM568F/RM2R for D3-4, RM2F/RM3R for D4-5, GA12F/RM4R for D5-6 and GA20F/RM9R for D8–9.

### Deep sequencing

Next, we performed MiSeq analysis of 18S or 28S rDNA amplicons using the two conventional and six newly selected primer sets. We used DNA extracted from the faeces of wild rats and a domesticated bovid as templates, which were anticipated to be highly rich in bacteria. Our previous morphological observations have revealed that five rats (i.e., WR4–8) were heavily infected with parasitic nematodes, while one rat (ZR4) was infected with tapeworms^4^. In contrast, the bovine sample (MB1) was rich in protozoan parasites. In total, 1,311,788 high-quality reads, with a mean of 23,425 reads per test (samples × primers), were obtained via Illumina MiSeq (Table S4).

**Figure 1:**
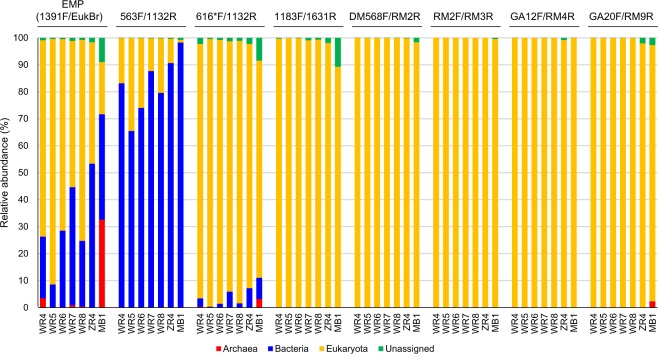
Domain-level classification of total Illumina reads retrieved from PCR amplicons using eight primer sets and seven faecal samples.

Taxonomic classification of the sequence reads revealed that EMP primer set (1391F/EukBr) amplicons contained numerous bacterial reads, with the highest observed in ZR4 (approximately 53%) and the lowest in WR5 (approximately 10%) (Fig. 1; Supplementary Table S5). 563F/1132R amplicons contained more bacterial reads than the EMP primer set amplicons for all samples. In particular, MB1 contained approximately 97% bacterial reads. 616F*/1132R amplicons contained fewer bacterial reads (<10%) than EMP primer set amplicons for all samples. The other primer set amplicons (i.e. 1183F/1631R, DM568F/RM2R, RM2F/RM3R, GA12F/RM4R and GA20F/RM9R) contained none or only a few bacterial reads (0–0.15%).

Archaea reads were detected in EMP, 616*F/1132R and GA20/RM9R amplicons, although few (<5%), except for MB1 amplicons of the EMP primer set which contained >30% Archaea reads. Relatively more unassigned reads (displaying no similarity with sequences in the database) were detected in MB1 amplicons of EMP, 616*F/1132R and 1183F/1631R primer sets (8.5%, 9.0% and 10.7%, respectively). Other combinations of primer sets and DNA samples revealed few unassigned reads (<2.2%).

**Figure 2:**
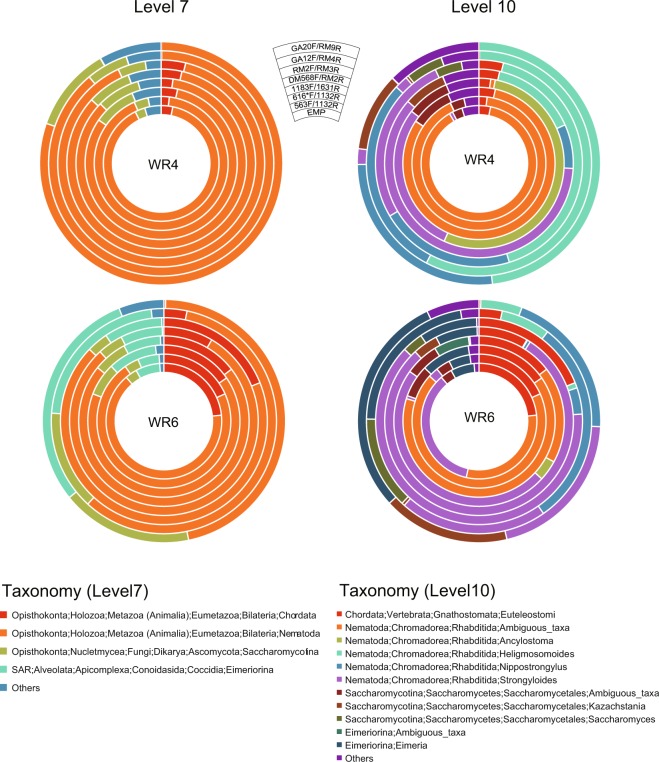
Taxonomic classification of eukaryotic reads in the faecal samples of nematode-infected rats (WR4 and WR6) at SILVA levels 7 and 10. Circles from the inside show the taxonomic distributions of reads obtained using the EMP (1391F/EukBr), 563F/1132R, 616*F/1132R, 1183F/1631R, DM568F/RM2F, RM2F/RM3R, GA12F/RM4R and GA20F/RM9R primer sets, respectively. Only taxa with ≥5% of the total non-bacterial reads are shown in the plots, and taxa with <5% are summarised as ‘Others’. Sequence reads without any taxonomic assignments because of low similarity with the database are shown as ‘Unassigned’.

**Figure 3:**
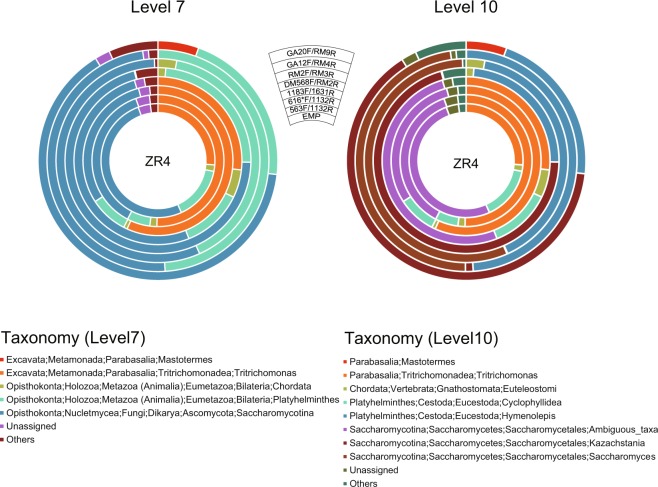
Taxonomic classification of eukaryotic reads in the faecal samples of a tapeworm-infected rat (ZR4) at SILVA levels 7 and 10. Circles from the inside show taxonomic distributions using the EMP (1391F/EukBr), 563F/1132R, 616*F/1132R, 1183F/1631R, DM568F/RM2F, RM2F/RM3R, GA12F/RM4R and GA20F/RM9R primer sets, respectively. Only taxa with ≥5% of the total non-bacterial reads are shown in the plots, and taxa with <5% are summarised as ‘Others’. Sequence reads without any taxonomic assignments because of low similarity with the database are shown as ‘Unassigned’.

**Figure 4:**
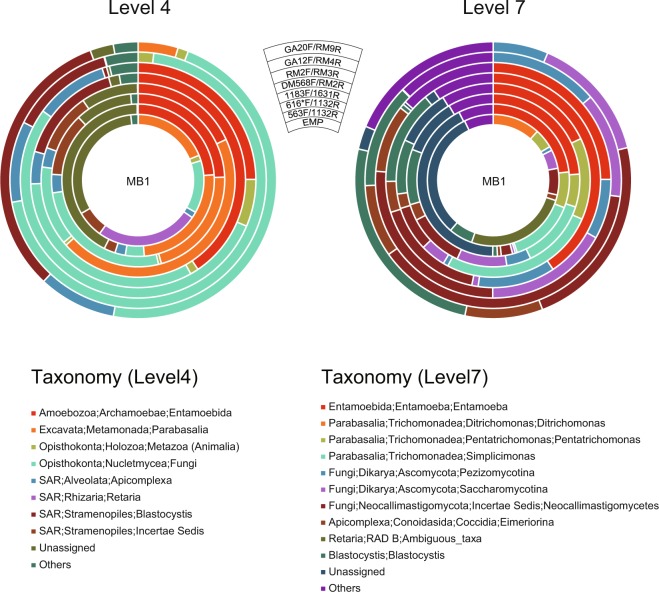
Taxonomic classification of eukaryotic reads in protozoa-rich bovine faecal samples (MB1) at SILVA levels 4 and 7. Circles from the inside show taxonomic distributions using the EMP (1391F/EukBr), 563F/1132R, 616*F/1132R, 1183F/1631R, DM568F/RM2F, RM2F/RM3R, GA12F/RM4R and GA20F/RM9R primer sets, respectively. Only taxa having ≥5% of the total non-bacterial reads are shown in the plots, and taxa with <5% are summarised in ‘Others’. Sequence reads without taxonomic assignments because of low similarity with the database are shown as ‘Unassigned’.

Numbers of operational taxonomic units (OTUs) detected using the EMP primer set ranged from 150 to 400 (Table S4). However, approximately half of the OTUs were assigned to either Bacteria or Archaea. Although the number of OTUs detected using 563F/1132R was the highest for each DNA sample (>1200 OTUs), after removing the bacteria and archaea reads, this number became the lowest among all primer sets. Other primer sets detected few or no bacterial OTUs, and the eukaryotic OTUs ranged from 40 to 500. Among these, RM2F/RM3R detected the lowest number of OTUs in the six rat samples, whereas GA12/RM4R detected the lowest numbers in the bovine sample. Overall, the six newly selected primer sets more readily avoided bacterial DNA amplification than the conventional primer sets. Finer classifications after removing the bacteria and archaea reads are shown in Figs 2–4.

### Nematode-infected samples

At the phylum level (SILVA level 7) classification, all primer sets exhibited similar taxon distribution patterns in WR4, although small proportional differences were noted (Fig. 2). Many reads (46–91%) were assigned to the phylum Nematoda and some were assigned to the phylum Chordata and sub-phylum Saccharomycotina using all primer sets. At SILVA level 10, Nematoda reads were further classified to the family or genus level. Using the three 18S primer sets (i.e. EMP, 563F/1132R and 616*F/1132R), many (>85%) Nematoda reads was assigned to ‘*Rhabditida; Ambiguous*’. Conversely, using 1183F/1631R, the proportion of ambiguous taxa became smaller and more reads were assigned to genera such as *Strongyloides* and *Ancylostoma*. Using the 28S primer sets, no ‘*Rhabditida; Ambiguous’* reads were detected and all Nematoda reads were subdivided into genera, including *Heligmosomoides*, *Nippostrongylus* and *Strongyloides*; this trend was similar to the nematode taxon distribution observed in our previous morphological identification^4^. Similar results were noted in other WR samples (i.e. WR5, WR7 and WR8), although differences in minor taxon distributions were observed (Supplementary Tables S7 and S8). In WR6, Eimeriorina was detected in addition to Nematoda, Chordata and Saccharomycotina using all primer sets (Fig. 2). At SILVA level 10, the Eimeriorina reads were further classified into *Eimeria* or ‘*Eimeriorina; Ambiguous*’ taxa.

### Tapeworm-infected samples

ZR4 harboured *Hymenolepis* tapeworms in its intestine. At SILVA level 7, all 18S primer sets detected high proportions of Platyhelminthes as well as Saccharomycotina and *Trichomonas* (or ‘*Trichomonas; Ambiguous*’) (Fig. 3; Supplementary Table S7). The three 28S primer sets did not detect *Trichomonas* reads, while GA20F/RM9R detected few *Trichomonas* reads (approximately 0.1%); therefore, Saccharomycotina and Platyhelminthes occupied higher proportions of the total reads using the 28S primer sets than using the 18S primer sets. Chordata reads were detected by all 18S primers and two 28S primer sets (i.e., DM568F/RM2R and RM2F/RM3R). *Mastotermes* was detected only by the GA20F/RM9R primer set. At SILVA level 10, Platyhelminthes reads were further classified to the order Cyclophyllidea using the 18S primer sets and to the genus *Hymenolepis* using the 28S primer sets. Saccharomycotina was further classified to the order Saccharomycetales using the 18S primer sets and to the genera *Saccharomyces* and *Kazachstania* using the 28S primer sets.

### Protozoa-rich samples

Various protozoa occur in the bovine gastrointestinal tract; thus, protozoal cysts are frequently detected in faecal samples. Most of these protozoa form a part of the normal ruminal microflora called ciliated protozoa^25,26^; however, some of these, such as *Eimeria* (*Coccidia*), *Cryptosporidium*, *Giardia*, *Entamoeba* and *Trichomonas*, are pathogenic and thus possess clinical significance^27^. In this study, we used bovine faeces as prototypical protozoa-rich samples.

At SILVA level 4, the EMP primer set (1391F/EukBr) detected reads assigned in descending order to Retaria, Parabasalia, Fungi, Stramenopiles, Apicomplexa and Metazoa (Fig. 4; Supplementary Table S6). Concomitantly, approximately 30% of the reads did not share similarities with known rDNA sequences in the database (unassigned). The other 18S primer sets produced similar patterns as the EMP primer sets. Approximately 10–40% of the reads were unassigned, and the remaining reads were primarily assigned to Parabasalia, Fungi, Apicomplexa and Stramenopiles. The largest differences from the EMP primer sets were Retaria and Entamoebida, which were detected only by the EMP and only by the other three 18S primer sets, respectively. The 28S primer sets detected lower proportions of unassigned reads than the 18S primer sets. The main detected taxa were similar between the 28S and 18S primer sets. However, Parabasalia reads were not detected by DM568F/RM2R, RM2F/RM3R and GA12F/RM4R, whereas Entamoebida reads were not detected by GA12F/RM4R and GA20F/RM9R. Instead of ‘*Stramenopiles; Incertae sedis*’, four 28S primer sets detected Stramenopiles; *Blastocystis*, although the proportion with RM2F/RM3R was minute.

At SILVA level 7, Parabasalia detected by the 18S primer sets were further classified into *Trichomonadea* taxa, including *Trichomonas*, *Ditrichomonas*, *Tetratrichomonas*, *Pentatrichomonas* and *Simplicimonas* (Fig. 4; Supplementary Table S7). Apicomplexa were further classified to Eimeriorina using all primer sets. Fungi were subdivided into the orders Neocallimastigomycetena, Saccharomycotina and Pezizomycotina, albeit without noticeable differences among the primer sets.

### Beta diversity analyses

**Figure 5:**
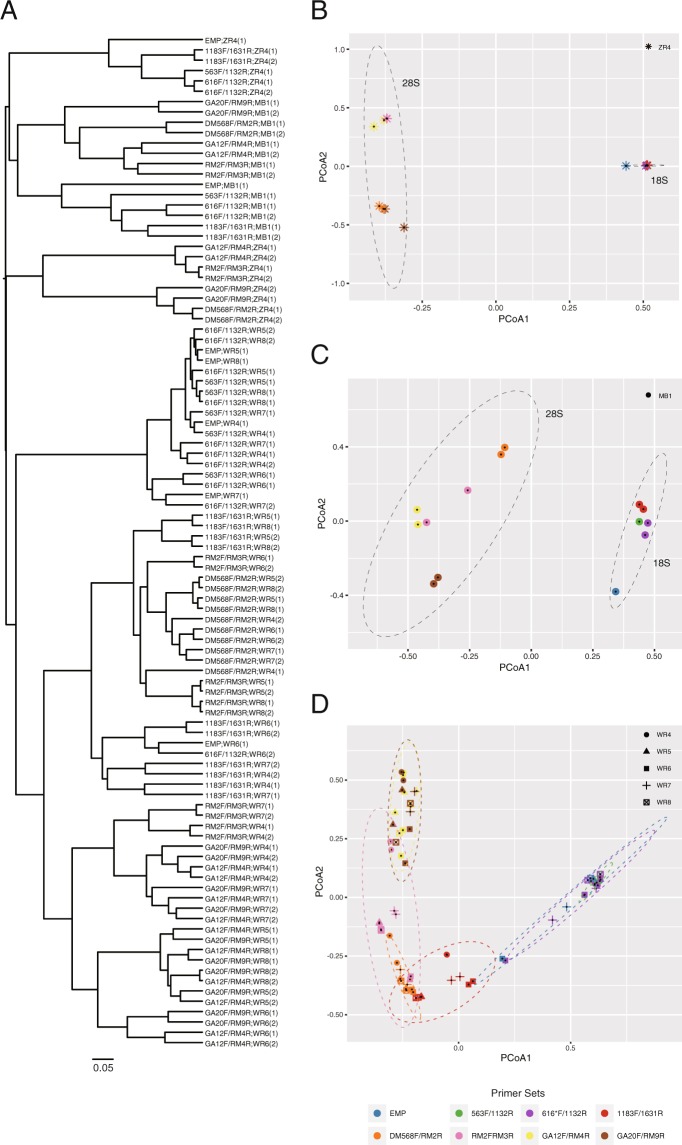
Cluster analysis and principal coordinate analysis (PCoA) of relative taxon abundance. (**A**) Dendrogram of cluster analysis based on the Bray–Curtis similarity index for the two replicate datasets. The labels on the edges represent ‘primer sets; DNA samples (dataset)’. (**B**–**D**) PCoA plots of ZR4, MB1 and WR samples, respectively. Ellipses drawn around the dots represent the 95% confidence limit for PCoA1 and 2 for 18S and 28S groups (**B**,**C**) or primer sets (**D**).

A technical replicate experiment was performed from PCR amplification to MiSeq independently from the first experiment using the newly selected primer sets (Dataset 2; Supplementary Tables S9–S12). The dendrograms of cluster analysis based on the Bray–Curtis dissimilarity of taxon abundance from the two replicate experiments are shown in Fig. 5A. All replicates (Datasets 1–2) in MB1 and ZR4 were clustered together in the dendrogram, suggesting high reproducibility of the methods using these primer sets (Fig. 5A). For WR samples, although the replicates were largely clustered together, some technical replicates were nested within the other DNA samples (e.g. WR4 with WR7 and WR5 with WR8), perhaps because those samples showed very similar taxonomic compositions. Principal coordinates analysis (PCoA) plots were generated for ZR4, MB1 and WR samples (Fig. 5B–D, respectively). In the three plots, PCoA1 separated the samples based on the PCR target regions (18S or 28S), although the separation in the WR plot, which contained five DNA samples, was not as obvious as that in the other plots. Among the 28S primer sets, RM2F/RM3R and GA12F/RM4R were clustered together in all the plots, whereas DM568F/RM2R and GA20F/RM4R were clustered together in the ZR and WR plots but not in the MB plot. Among the 18S primer sets, 563F/1132R and 616*/F1132R were clustered together in all the plots. These results correspond to the target regions of 18S or 28S (Tables 1 and 2).

## Discussion

Bacterial read contamination of PCR amplicons often poses a critical problem in NGS-based analyses of eukaryotic diversity or diagnoses. In extreme cases, as with MiSeq of a bovine faecal sample in the present study, over 95% of the total sequence reads can be derived from bacterial DNA, making it difficult to detect rare eukaryotes in the samples. Increasing data acquisition may resolve this issue; however, presence of raw data with only one or two orders of magnitude than non-contaminated cases is inefficient and therefore prevents high-throughput analyses. The primer sets newly screened in this study, which can efficiently amplify rDNA from a wide range of eukaryotes without bacterial DNA amplification, are anticipated to be suitable tools for diversity analyses of eukaryotic microbes, including parasites.

At the same time, we noted that each primer set could not detect a specific taxonomy groups. According to our deep sequencing analysis, two of the 28S primer sets could not to detect *Trichomonas* species, which were detected by all other 18S primer sets. *Spironucleus* reads were detected from rat faeces using only one 28S primer set (GA20F/RM9R). *Entamoeba* could not be detected using the EMP and GA20F/RM9R primer sets. In addition, the results of *in silico* analysis suggested that one primer set is unlikely to cover all taxonomic groups of parasites. For instances, *Plasmodium* spp., one of the most medically important parasites, was difficult to detect using any of the tested 18S rDNA primer sets, although it may be detected using the two 28S rDNA primer sets. *Trypanosoma* and *Leishmania*, two other important parasitic genera, could be detected only using 563F/1132R and 1183F/1631R among the tested primers. Collectively, these results suggest the importance of selecting primer sets according to the study objective.

To achieve fine taxonomic resolution, long sequences containing sufficient diversity to distinguish closely related species are essential. Although sequencing technologies capable of producing long sequences, such as PacBio and NanoPore, are available^28,29^, these remain impractical for rDNA-based microbiome analyses because of their higher error rates and lower throughputs than those of Illumina sequencing. Therefore, many studies have used Illumina sequencing, for which the maximum length is 600 bp (300-bp paired-end). Although we used variable regions of 18S rDNA with fragment lengths ranging from 150 to 570 for taxonomic classification, we were unable to further assign the reads to the genus or species level in most cases. On the contrary, reads of 28S rDNA, which has higher sequence diversity than 18S rDNA^13^, could sometimes be further assigned to the genus level, suggesting that 28S rDNA represents a good option for studies in which finer classification is necessary. One of the challenges in 28S rDNA-based population analyses is the enlargement of the database because database sizes affect fine taxonomic classification. The current database (SILVA r132) contains 198,843 28S rDNA sequences compared with 695,171 18S rDNA sequences (https://www.arb-silva.de/). In addition, we discarded primer sets with amplicon sizes that were out of range even though they demonstrated good taxonomic coverages (Supplementary Table S13). These primers can be used as alternates if they are capable of amplifying sequences to meet the length requirement.

Host DNA contamination did not hamper analyses in this study. Small proportions of mammalian (Chordata) reads were detected with any combination of samples and primers. This is probably because the faecal samples used in this study were collected from wild animal and contained high number of eukaryotic microbes. However, our *in-silico* analysis revealed that all the tested primer sets theoretically cannot avoid amplification of host DNA. Therefore, when samples are expected to have small amounts of eukaryotic microbes, such as clinical samples from human or samples from well-kept pets, PCR blockers may be required, which prevent host DNA amplification^30–32^. Applying taxon-specific primers is an alternative option to avoid amplification of host DNA. Recently, Cannon *et al*.^33^ proposed a high-throughput method to detect a wide range of parasites by a combination of multiple taxon-specific primers. We tested those primers using our evaluation criteria and confirmed that those primer sets amplify each targeted taxa and can avoid host and bacterial DNA amplification (Table S14). Although this strategy requires optimisation for multiplex PCR (amplification of multiple targets in a single PCR) for high throughput studies and may require a reasonable normalisation method for amplification bias by each primer set for a reliable estimation of taxa distribution in a sample, the assay still has an advantages in customizability to easily include additional targeted taxa^33^. Therefore, the primer sets selected in this study can be added to the multiplex assay, which could achieve more comprehensive “parasitome” analyses.

The benefits and drawbacks of the newly selected primer sets and conventional primers are summarised in Table 3. First, the newly selected primer sets could avoid bacterial DNA amplification. However, taxonomic coverage differed with each primer set. Ultimately, the primer sets should be selected according to the study objectives, taking the parasites that need to be covered and the required resolution into account. However, we recommend the use of 616*/F1132R for 18S rDNA or DM568F/RM2R for 28S rDNA, or a combination of those, as new standard primer sets for parasite detection because these provide wide taxonomic coverage of parasitic eukaryotes with minimal bacterial DNA contamination.

**Table 3:** A summary of 18S and 28S rDNA primer set evaluation.

	EMP (1391F/EukBr)	563F/1132R	616*F/1132R	1183F/1631R	DM568F/RM2R	RM2F/RM3R	GA12F/RM4R	GA20F/RM9R
rDNA	18S	18S	18S	18S	28S	28S	28S	28S
Target variable region	V9	V4–5	V4–5	V7–8	D4–5	D4–5	D4–6	D8–9
Degeneracy (forward/reverse)	0/0	3/3	4/3	0/0	0/1	1/1	0/5	1/1
Amplicon size (bp)^a^	145	569	516	449	284	236	507	505
Bacterial contamination	−	−−	+	++	++	++	++	++
**Taxonomic coverage** ^**b**^								
Nematoda	**+**	**++**	**++**	**++**	**+**	**++**	**++**	**++**
Platyhelminthes	**+**	**++**	**++**	**++**	**+**	**++**	**++**	**++**
Acanthocephala	**+**	**++**	**++**	**−**	**++**	**++**	**++**	**++**
Coccidia	**+**	**++**	**++**	**++**	**++**	**++**	**++**	**++**
Cryptosporida	**+**	**++**	**++**	**++**	**++**	**++**	**++**	**++**
Haemosporidia	**+**	**−**	**−**	**−**	**++**	**++**	**−**	**++**
Fornicata	**+**	**++**	**+**	**+**	**+**	**−**	**−**	**++**
Discicristata	**+**	**++**	**+**	**++**	**+**	**+**	**++**	**+**
Parabasalia	**+**	**++**	**+**	**−**	**++**	**−**	**+**	**++**
Entamoebida	**+**	**++**	**++**	**−**	**++**	**−**	**−**	**−**
Longamoebia	**+**	**++**	**++**	**++**	**++**	**++**	**++**	**++**

^a^Based on *S. cerevisiae* rRNA gene (NC_001144).

^b^++; >85%, +; 10–85%, −; <10% in the *in silico* taxonomy coverage test. Bold characters indicate detections confirmed using MiSeq sequencing in this study.

## Methods

### SSU and LSU primer screening

Potential universal primer sequences targeting eukaryote rDNA were obtained from previous studies^5,6,13,23,24^. The primers were filtered to select primer pairs suitable for Illumina MiSeq analysis under the following criteria: Tm in the range of 55 °C–70 °C, a difference in Tm between the two primers of <5 °C and an amplicon size of 200–580 bp. These primer pairs were further evaluated for similarities with eukaryote and bacterial rDNA sequences using TestPrime 1.0 and the SILVA 132 database under the following parameters: maximum number of mismatch = 4 bp and the length of 0-mismatch zone at the 3′ end = 3 bp). We used the non-redundant reference dataset (Ref NR) build by a dereplication of the full reference set using a 99% identity criterion and were suggested by SILVA to be used as a representative dataset for classification, phylogenetic analysis and probe design.

### DNA samples

A bacterial DNA mixture was prepared by combining 70 ng DNA extracted from pure cultures of seven bacterial species (*Escherichia coli*, *Enterobacter* sp., *Serratia* sp., *Bacillus subtilis*, *Klebsiella pneumoniae*, Group A *Streptococcus* and *Staphylococcus epidermidis*) using a QIAmp DNA Mini Kit (Qiagen). *C. elegans* DNA was extracted from approximately 10,000 worms using the same kit.

For MiSeq analyses, DNA extracted from the faeces of rats caught in the Miyazaki City Phoenix Zoo (ZR, *Rattus rattus*) or in Miyazaki downtown (WR, *Rattus norvegicus*) in our previous study^4^ were used. Faecal samples from a domesticated bovid (MB, *Bos taurus*) were provided by the veterinary parasitology lab of the University of Miyazaki, and DNA was extracted using a Maxwell RSC Purefood GMO Kit (Promega), as described previously^34^.

### qPCR

qPCR was performed to test the amplification efficiency of each primer set using *C. elegans* DNA or the bacterial DNA mixture as a template. Reactions were performed in triplicates using a StepOnePlus Real-Time PCR System (Applied Biosystems) under the following conditions: 95 °C for 10 min, followed by 40 cycles of 95 °C for 15 s, 50 °C for 30 s and 60 °C for 1 min (for 18S rDNA amplification), or 95 °C for 10 min, followed by 40 cycles of 95 °C for 15 s and 60 °C for 1 min (for 28S rDNA amplification). The reaction volume was 10 μl, including 5 μl of the Power SYBR Green PCR Master Mix (2x), 0.9 μM of each primer and 1 μl of DNA solution. To calculate the PCR efficiencies and detection limits, serial 10-fold dilutions of *C. elegans* DNA (1 ng to 0.01 pg) were used as templates.

### MiSeq sequencing

PCR was performed using Tks Gflex DNA Polymerase (Takara), and a 30-µl reaction mixture containing 1 µl of template DNA (1–3 ng of DNA), 15 µl of 2 × Gflex buffer, 0.5 µl each of the forward/reverse primers with the Illumina MiSeq Adapter (10 µM final concentration), 0.5 µl (100 U) of DNA polymerase and 13 µl of nuclease-free H_2_O. Reactions were performed using Veriti Thermal Cycler (Applied Biosystems) under the following conditions: 95 °C for 1 min, followed by 35 cycles of 95 °C for 15 s, 60 °C (for 28S rDNA amplification) or 50 °C (18S rDNA amplification) for 1 min and 68 °C for 1 min. Duplicate PCRs were performed independently, and the produced materials were then mixed. The PCR products were confirmed via agarose gel electrophoresis and purified using AMpure XP beads (Beckman Coulter). Index PCR was performed to attach dual indices and Illumina sequencing adapters to the first PCR products using the Nextera XT Index Kit (Illumina) and KAPA HiFi HotStart Ready Mix (Kapa Biosystems) under the following conditions: 95 °C for 3 min, followed by 8 cycles of 95 °C for 30 s, 55 °C for 30 s and 72 °C for 30 s, and the final extension at 72 °C for 5 min. The PCR product was cleaned using AMpure XP beads, pooled at equal concentrations and then sequenced using the MiSeq Reagent Nano Kit v3 (600 cycles) according to the manufacturer’s protocol (http://icom.illumina.com/) to produce 300-bp paired-end reads.

### Bioinformatic analysis

Illumina sequence data were processed using QIIME version 1.9.1^35^. Paired-end reads were joined using the ‘fastq-join’ method (join_paired_ends.py). After QIIME quality filtering (split_libraries_fastq.py: -store_qual_scores -q 9 -max_barcode_errors 2 -sequence_max_n 1 -max_bad_run_length 2 -p 0.5 –r 3), chimeric sequences were detected using the UCHIME algorithm, which is included in the free version of USEARCH61, and eliminated from further analyses. Cleaned reads were clustered and assigned to OTUs using the open-reference OTU-picking protocol with the SILVA 128 database^36^ at 97% identity with ‘blast’ (pick_open_reference_otus.py).

Similarity in taxa composition and the relative abundance were analysed via PCoA and hierarchical cluster analyses using the Bray–Curtis similarity index with R vegan package^37^.

## Supplementary information

Supplementary information

Supplementary information

## Data Availability

The sequencing data have been deposited to the DNA Data Bank of Japan Sequence Read Archive under the BioProject PRJDB3050.
