# Supplementary material for: Improved 18S and 28S rDNA primer sets for NGS-based parasite detection
Source: Sci Rep. 2019 Oct 31;9:15789. doi: 10.1038/s41598-019-52422-z (PMC6823512; doi:10.1038/s41598-019-52422-z)
Supplement: Supplementary file 1 — Supplementary information [file 41598_2019_52422_MOESM1_ESM.pdf]

Supplementary information for

Improved 18S and 28S rDNA primer sets for NGS-based parasite detection

Asuka Kounosu, Kazunori Murase, Akemi Yoshida, Haruhiko Maruyama and Taisei Kikuchi*

Table S1. List of 18S and 28S rDNA primers evaluated in this study.

18S rDNA primers

Primer ID	Position[1]	Sequence	Tm (˚C)	CG%	Referenece
1391F	1629	GTA CAC ACC GCC CGT C	61.9	68.8	Thorsten et.al (2010)
EukbR	1774	TGA TCC TTC TGC AGG TTC ACC TAC	67.6	50.0	Thorsten et.al (2010)
563F	563	GCC AGC AVC YGC GGT AAY	68.4	64.8	Hugerthet.al (2014)
574F	574	CGG TAA YTC CAG CTC YAV	58.6	53.7	Hugerthet.al (2014)
574*F	574	CGG TAA YTC CAG CTC YV	58.8	56.9	Hugerthet.al (2014)
616*F	616	TTA AAR VGY TCG TAG TYG	53.8	39.8	Hugerthet.al (2014)
1132R	1150	CCG TCA ATT HCT TYA ART	53.9	35.2	Hugerthet.al (2014)
566F	566	CAG CAG CCG CGG TAA TTC C	70.2	63.2	Hadziavdic et.al (2014)
952R	952	TTG GCA AAT GCT TTC GC	64.1	47.1	Hadziavdic et.al (2014)
1183F	957	AAT TTG ACT CAA CAC GGG	59.1	44.4	Hadziavdic et.al (2014)
1200R	1200	CCC GTG TTG AGT CAA ATT AAG C	64.7	45.5	Hadziavdic et.al (2014)
1631R	1246	TAC AAA GGG CAG GGA CGT AAT	64.6	47.6	Hadziavdic et.al (2014)

28S rDNA primers

Primer ID	Position[1]	Sequence	Tm (°C)	%GC	Referenece
RM1F	636	CCG TCT TGA AAC ACG GDC YRA G	70.1	56.1	Machida et.al (2012)
RM2F	905	AGG GGC GAA AGA CYA ATC GAA	68.5	50	Machida et.al (2012)
RM2R	925	TTC GAT TRG TCT TTC GCC CCT	68.5	50	Machida et.al (2012)
RM3F	1121	TTT TGG TAA GCA GAA CTG GYG	64.3	45.2	Machida et.al (2012)
RM3R	1141	CRC CAG TTC TGC TTA CCA AAA	64.3	45.2	Machida et.al (2012)
RM4F	1434	GAT CTY RGT GGY AGT AGC RAV T	61.7	48.5	Machida et.al (2012)
RM4R	1454	ABT YGC TAC TRC CAC YRA GAT C	61.7	48.5	Machida et.al (2012)
RM5F	2121	GGG AAT CYR ACT GTH TAA TTA AA	58.3	31.9	Machida et.al (2012)
RM5R	2143	TTT AAT TAD ACA GTY RGA TTC CC	58.3	31.9	Machida et.al (2012)
RM6F	2185	TGA TTT CTG CCC AGT GCT YWG AAW GT	71	44.2	Machida et.al (2012)
RM6R	2210	ACW TTC WRA GCA CTG GGC AGA AAT CA	71	44.2	Machida et.al (2012)
RM7F	2242	AAC GGC GGR RGT AAC TAT GAC TYT	67.2	47.9	Machida et.al (2012)
RM7R	2265	ARA GTC ATA GTT ACY YCC GCC GTT	67.2	47.9	Machida et.al (2012)
RM8F	2393	GGG AAA GAA GAC CCT GTT GAG	64.2	52.4	Machida et.al (2012)
RM8R	2413	CTC AAC AGG GTC TTC TTT CCC	64.2	52.4	Machida et.al (2012)
RM9F	2401	AAG ACC CTG TTG AGY TTG ACT CT	63.6	45.7	Machida et.al (2012)
RM9R	2422	AGA GTC AAR CTC AAC AGG GTC TT	63.6	45.7	Machida et.al (2012)
RM10F	2605	GGG AGT TTG RCT GGG GCG G	73.3	71.1	Machida et.al (2012)
RM10R	2623	CCG CCC CAG YCA AAC TCC C	73.3	71.1	Machida et.al (2012)
RM11R	2836	GCT TGG CBG CCA CAA GCC AGT TA	75.3	59.4	Machida et.al (2012)
GA4F	26	ACC CGC TGA AYT TAA GCA TAT	61.7	40.5	Auwera et.al (1993)
GA5R	46	ATA TGC TTA ART TCA GCG GGT	61.7	40.5	Auwera et.al (1993)
GA6F	339	CGA TAG YVR ACA AGT A	46.9	41.7	Auwera et.al (1993)
GA7R	354	TAC TTG TYB RCT ATC G	46.9	41.7	Auwera et.al (1993)
GA8F	643	ATC TTG GTG GAC GAG T	53.8	50	Auwera et.al (1993)
GA9R	658	ACT CGT CCA CCA AGA T	53.8	50	Auwera et.al (1993)
GA10F	636	CCG TCT TGA AAC ACG GAC CAA GGA G	74.1	56	Auwera et.al (1993)
GA11R	660	CTC CTT GGT CCG TGT TTC AAG ACG G	74.1	56	Auwera et.al (1993)
GA12F	947	CCG AAG TTT CCC TCA GGA TAG C	57.9	47.4	Auwera et.al (1993)

GA13R	968	GCT ATC CTG AGG GAA ACT TCG G	67.1	54.5	Auwera et.al (1993)
GA14F	1252	TCC GCT AAG GAG TGT GTA ACA AC	64.4	47.8	Auwera et.al (1993)
GA15R	1274	GTT GTT ACA CAC TCC TTA GCG GA	64.4	47.8	Auwera et.al (1993)
GA16F	1519	GGT YAG TCG RTC CTR AG	54.4	55.9	Auwera et.al (1993)
GA17R	1536	TCT YAG GAY CGA CTN AC	52.2	50	Auwera et.al (1993)
GA18F	1841	CCG CAK CAG GTC TCC AA	65.6	61.8	Auwera et.al (1993)
GA19R	1858	CTT GGA GAC CTG MTG CGG	66.4	63.9	Auwera et.al (1993)
GA20F	1917	GTA ACT TCG GGA WAA GGA TTG GCT	67.1	45.8	Auwera et.al (1993)
GA21R	1940	AGC CAA TCC TTW TCC CGA AGT TAC	67.1	45.8	Auwera et.al (1993)
GA22F	2185	TGA TTT CTG CCC AGT GCT CTG AAT GT	72.5	46.2	Auwera et.al (1993)
GA23R	2210	ACA TTC AGA GCA CTG GGC AGA AAT CA	72.5	46.2	Auwera et.al (1993)
GA24F	2393	GGG AAA GAA GAC CCT GTT GAG	64.2	52.4	Auwera et.al (1993)
GA25R	2413	CTC AAC AGG GTC TTC TTT CC	60.8	50	Auwera et.al (1993)
GA26F	2605	GGG AGT TTG RCT GGG GCG G	73.3	71.1	Auwera et.al (1993)
GA27R	2623	CCG CCC CAG YCA AAC TCC C	73.3	71.1	Auwera et.al (1993)
GA28F	2932	AGG GAA CGT GAG CTG GGT TTA GAC	69.4	54.2	Auwera et.al (1993)
GA29R	2955	GTC TAA ACC CAG CTC ACG TTC CCT	69.4	54.2	Auwera et.al (1993)
GA30F	3106	CTG AAC GCC TCT AAG TCA GAA	61.6	47.6	Auwera et.al (1993)
GA31R	3126	TTC TGA CTT AGA GGC GTT CAG	61.6	47.6	Auwera et.al (1993)
DM1F	26	ACC CGC TGA ATT TAA GCA T	61.5	42.1	Moreira et.al (2006)
DM2F	1430	GCA GAT CTT GGT GGT AG	54.3	52.9	Moreira et.al (2006)
DM3R	1861	CAC CTT GGA GAC CTG CT	59.5	58.8	Moreira et.al (2006)
DM4R	3126	TTC TGA CTT AGA GGC GTT CAG	61.6	47.6	Moreira et.al (2006)
DM568F	641	TTG AAA CAC GGA CCA AGG AG	65.4	50	Moreira et.al (2006)
DM1611R	1858	CTT GGA SAC CTG MTG CGG	66.8	63.9	Moreira et.al (2006)

[1] positions of primer 5′ end in *S. cerevisiae* rRNA gene (NC_001144)

Table S2. Primer sets targeting the 18S or 28S rRNA gene and their coverages in 13 taxonomic groups under a strict TestPrime parameter set (maximum number of mismatches of three bases and length of 0-mismatch at the 3’ end of three bases)

Taxonomy	Representative species	1391F/EukBr[1,2]		563F/1132R[2]		574F/952R[2]		574*F/952R[2]		616*F/1132R[2]		1183F/1631R[2]	
		V9		V4-V5		V4-V5		V4-V5		V4-V5		V7-V8	
		145 bp		569 bp		378 bp		378 bp		516 bp		449 bp	
Nematoda	Roundworm, Filaria	88.0	(550/625)	95.9	(2072/2160)	42.0	(908/2163)	93.1	(2014/2163)	96.0	(2080/2166)	87.9	(1822/2072)
Platyhelminthes	Tapeworm, Fluke	81.3	(304/374)	88.3	(1710/1937)	88.7	(1725/945)	88.4	(1719/1945)	88.5	(1724/1948)	90.1	(1712/1900)
Acanthocephala	Spiny-headed worm	100.0	(26/26)	95.2	(60/63)	0.0	(0/63)	0.0	(0/63)	93.7	(59/63)	0.0	(0/62)
Coccidia	Coccidium	95.4	(167/175)	97.9	(656/670)	96.6	(648/671)	96.6	(648/671)	97.2	(652/971)	90.8	(444/489)
Cryptosporida	Cryptosporidium	97.3	(36/37)	94.5	(52/55)	96.4	(53/55)	96.4	(53/55)	92.7	(51/55)	98.1	(52/53)
Haemosporidia	Plasmodium	87.5	(63/72)	0.0	(0/148)	98.0	(145/148)	98.0	(145/148)	0.0	(0/149)	0.0	(0/95)
Fornicata	Giardia	100.0	(7/7)	100.0	(21/21)	23.8	(5/21)	33.3	(7/21)	71.4	(15/21)	30.0	(6/20)
Discicristata	Trypanosoma, Leishmania	91.9	(305/332)	92.7	(804/867)	37.2	(333/894)	37.4	(334/893)	71.4	(638/893)	85.8	(695/810)
Parabasalia	Trichomonas	77.0	(47/61)	98.0	(297/303)	0.0	(0/329)	13.2	(40/303)	19.5	(59/303)	0.0	(0/299)
Entamoebida	Entamoeba	100.0	(3/3)	100.0	(4/4)	0.0	(0/4)	0.0	(0/4)	100.0	(4/4)	0.0	(0/3)
Longamoebia	Acanthamoeba	67.9	(36/53)	86.0	(123/143)	88.4	(129/146)	89.7	(131/146)	88.0	(132/150)	88.0	(117/133)
Fungi		56.1	(3064/5458)	96.4	(14469/15009)	93.7	(14123/15071)	94.2	(14192/15071)	93.7	(14142/16036)	91.7	(13100/14290)
Bacteria		36.2	(32718/90407)	89.7	(604569/663741)	<0.1	(12/575261)	<0.1	(12/575261)	<0.1	(12/575050)	<0.1	(1/558087)

DM568F/RM2R[2]		DM568F/GA13R[2]		DM568F/RM3R[2]		RM2F/RM3R[2]		RM2F/GA15R[2]		GA12F/RM4R[2]		RM3F/RM4R[2]		GA14F/RM4R[2]		GA18F/RM7R[2]	
D3-D4		D3-D4		D3-D5		D4-D5		D4-D5		D4-D6		D6		D6		D8	
284 bp		327 bp		500 bp		236 bp		369 bp		507 bp		333 bp		202 bp		424 bp	
41.8	(228/546)	2.8	(123/4346)	41.8	(228/546)	94.7	(551/582)	54.8	(319/582)	92.2	(543/589)	91.5	(558/610)	90.2	(586/650)	99.8	(625/653)
88.2	(439/498)	84.7	(422/498)	91.4	(455/498)	99.5	(4951/4977)	23.2	(121/521)	97.7	(512/524)	97.6	(522/535)	96.3	(524/544)	86.3	(465/539)
94.6	(53/56)	229.4	(39/17)	94.6	(53/56)	94.6	(53/56)	28.6	(16/56)	94.6	(53/56)	94.6	(53/56)	35.7	(20/56)	96.4	(54/56)
90.4	(66/73)	91.8	(67/73)	90.4	(66/73)	91.9	(68/74)	82.3	(65/79)	93.2	(69/74)	91.9	(68/74)	91.9	(68/74)	97.1	(68/70)
100.0	(1/1)	100.0	(1/1)	100.0	(1/1)	100.0	(1/1)	100.0	(1/1)	100.0	(1/1)	100.0	(1/1)	100.0	(1/1)	100.0	(1/1)
100.0	(89/89)	0.0	(0/89)	95.5	(85/89)	94.9	(93/98)	87.8	(86/98)	0.0	(0/101)	94.2	(98/104)	94.5	(103/109)	61.1	(69/113)
20.0	(1/5)	0.0	(0/5)	0.0	(0/5)	0.0	(0/5)	20.0	(1/5)	20.0	(1/5)	0.0	(0/5)	20.0	(1/5)	0.0	(0/5)
67.7	(21/31)	61.3	(19/31)	41.9	(13/31)	40.6	(13/32)	75.0	(24/32)	75.8	(25/33)	51.4	(18/35)	77.1	(27/35)	48.6	(17/35)
82.4	(14/17)	82.4	(14/17)	0.0	(0/17)	0.0	(0/18)	22.2	(4/18)	0.0	(0/18)	0.0	(0/20)	0.0	(0/20)	0.0	(0/20)
100.0	(5/5)	0.0	(0/5)	0.0	(0/5)	0.0	(0/8)	0.0	(0/8)	0.0	(0/8)	0.0	(9/9)	100.0	(9/9)	0.0	(0/9)
100.0	(1/1)	100.0	(1/1)	100.0	(1/1)	100.0	(1/1)	100.0	(1/1)	100.0	(1/1)	100.0	(1/1)	100.0	(1/1)	100.0	(1/1)
81.1	(2852/3515)	92.9	(3267/3515)	93.4	(3283/3515)	93.4	(3340/3576)	85.0	(3041/3576)	95.5	(3419/3579)	94.8	(3423/3612)	95.2	(3401/3571)	90.4	(2934/3244)
<0.1	(9/122777)	<0.1	(9/122777)	<0.1	(9/122777)	<0.1	(9/123872)	<0.1	(8/123872)	<0.1	(5/124050)	<0.1	(5/124626)	<0.1	(6/124788)	<0.1	(13/124775)

GA18F/RM8R[2]		GA20F/RM7R[2]		GA20F/RM8R[2]		GA20F/RM9R[2]	
D8		D8		D8-D9		D8-D9	
572 bp		348 bp		406 bp		505 bp	
95.8	(619/646)	97.5	(637/653)	97.8	(632/646)	10.1	(632/6245)
44.8	(47/105)	91.3	(492/539)	94.1	(498/529)	96.2	(508/528)
100.0	(56/56)	82.1	(46/56)	85.7	(48/56)	85.7	(48/56)
95.5	(64/67)	98.6	(69/70)	97.0	(65/67)	98.5	(65/66)
100.0	(1/1)	100.0	(1/1)	100.0	(1/1)	100.0	(1/1)
61.6	(69/112)	92.0	(104/113)	97.3	(109/112)	97.3	(109/112)
0.0	(0/5)	60.0	(3/5)	60.0	(3/5)	60.0	(3/5)
24.6	(16/35)	48.6	(17/35)	23.5	(16/68)	48.6	(17/35)
36.8	(7/19)	0.0	(0/20)	94.7	(18/19)	94.7	(18/19)
0.0	(0/9)	0.0	(0/9)	0.0	(0/9)	0.0	(0/9)
100.0	(1/1)	100.0	(1/1)	100.0	(1/1)	100.0	(1/1)
88.3	(2830/3205)	95.4	(3059/3244)	92.6	(2969/3205)	92.0	(2984/3205)
<0.1	(10/124065)	<0.1	(13/124775)	<0.1	(10/124065)	<0.1	(10/124031)

Primer sets were tested for matches to sequences in the SILVA database (v.132) using TestPrime with the parameters (maximum number of mismatches of three bases and length of 0-mismatch at the 3′ end of three bases). TestPrime computes coverages for each taxonomic group by running *in silico* PCR on the SILVA database via sorting database sequences into “match”, “mismatch” and “nodata (sequences not covering the primer match position)”. The frequencies of “match” sequences among “match” and “mismatch” sequences are shown as percentages with the sequence numbers in parentheses.

[1] Only 17 bases from the 3’ was used for the primer EukBr because many sequences in the SILVA database lacks the corresponding 5’ region.

[2] Target variable regions and amplicon sizes based on the *S. cerevisiae* rRNA gene (NC_001144) are shown below the primer names.

Table S3. Primer sets targeting the 18S or 28S rRNA gene and their coverages in 13 taxonomic groups under a mild TestPrime parameter set (maximum number of mismatches of five bases and length of 0-mismatch at the 3’ end of three bases)

Taxonomy	Representative species	1391F/EukBr[1,2]		563F/1132R[2]		574F/952R[2]		574*F/952R[2]		616*F/1132R[2]		1183F/1631R[2]	
		V9		V4-V5		V4-V5		V4-V5		V4-V5		V7-V8	
		145 bp		569 bp		378 bp		378 bp		516 bp		449 bp	
Nematoda	Roundworm, Filaria	88.3	(552/625)	95.9	(2072/2160)	42.0	(909/2163)	93.2	(2016/2163)	96.0	(2080/2166)	88.1	(1384/1631)
Platyhelminthes	Tapeworm, Fluke	82.1	(307/374)	88.4	(1713/1937)	89.2	(1735/1945)	88.9	(1730/1946)	88.7	(1727/1947)	90.2	(1715/1901)
Acanthocephala	Spiny-headed worm	100.0	(26/26)	95.2	(60/63)	0.0	(0/63)	0.0	(0/63)	93.7	(59/63)	0.0	(0/65)
Coccidia	Coccidium	96.0	(168/175)	97.9	(656/670)	96.7	(649/671)	96.7	(649/671)	97.2	(652/671)	90.8	(444/489)
Cryptosporida	Cryptosporidium	97.3	(36/37)	94.5	(52/55)	96.4	(53/55)	96.4	(53/55)	92.7	(51/55)	100.0	(53/53)
Haemosporidia	Plasmodium	87.5	(63/72)	0.0	(0/148)	98.0	(145/148)	98.0	(145/148)	0.0	(0/149)	0.0	(0/95)
Fornicata	Giardia	100.0	(7/7)	100.0	(21/21)	33.3	(7/21)	42.9	(9/21)	71.4	(15/21)	30.0	(6/20)
Discicristata	Trypanosoma, Leishmania	92.2	(306/332)	93.0	(806/867)	81.3	(727/894)	84.8	(736/894)	71.7	(640/893)	86.3	(699/810)
Parabasalia	Trichomonas	77.0	(47/61)	98.0	(297/303)	0.0	(0/303)	82.8	(251/303)	19.8	(60/303)	0.0	(0/299)
Entamoebida	Entamoeba	100.0	(3/3)	100.0	(4/4)	100.0	(4/4)	100.0	(4/4)	100.0	(4/4)	0.0	(0/3)
Longamoebia	Acanthamoeba	73.6	(39/53)	87.4	(125/125)	89.0	(130/146)	90.4	(132/146)	88.0	(132/150)	89.5	(119/133)
Fungi		56.4	(3078/5458)	96.8	(14519/15006)	95.5	(14388/15071)	95.9	(14460/15070)	93.8	(14160/15100)	92.0	(13148/14290)
Bacteria		37.9	(34245/90407)	90.0	(517733/575261)	<0.1	(14/575261)	<0.1	(40/575261)	<0.1	(258/575265)	<0.1	(1/558088)

Taxonomy	DM568F/RM2R[2]		DM568F/GA13R[2,3]	DM568F/RM3R[2]		RM2F/RM3R[2]		RM2F/GA15R[2]		GA12F/RM4R[2,3]	RM3F/RM4R[2]	
	D3-D4		D3-D4	D3-D5		D4-D5		D4-D5		D4-D6	D6	
	284 bp		327 bp	500 bp		236 bp		369 bp		507 bp	333 bp	
Nematoda	62.1	(339/546)		62.1	(339/546)	64.0	(55/86)	54.8	(319/582)		92.5	(564/610)
Platyhelminthes	88.4	(440/498)		91.4	(455/498)	95.4	(497/521)	23.4	(122/521)		97.8	(523/535)
Acanthocephala	94.6	(53/56)		94.6	(53/56)	94.6	(53/56)	28.6	(16/56)		100.0	(56/56)
Coccidia	90.4	(66/73)		90.4	(66/73)	91.9	(68/74)	83.8	(62/74)		91.9	(68/74)
Cryptosporida	100.0	(1/1)		100.0	(1/1)	100.0	(1/1)	100.0	(1/1)		100.0	(1/1)
Haemosporidia	100.0	(89/89)		95.5	(85/89)	94.9	(93/98)	87.8	(86/98)		95.2	(99/104)
Fornicata	20.0	(1/5)		0.0	(0/5)	0.0	(0/5)	20.0	(1/5)		20.0	(1/5)
Discicristata	67.7	(21/31)		41.9	(13/31)	40.6	(13/32)	75.0	(24/32)		85.7	(30/35)
Parabasalia	63.0	(17/27)		0.0	(0/17)	0.0	(0/18)	61.1	(11/18)		5.0	(1/20)
Entamoebida	100.0	(5/5)		0.0	(0/5)	0.0	(0/8)	0.0	(0/8)		100.0	(9/9)
Longamoebia	100.0	(1/1)		100.0	(1/1)	100.0	(1/1)	100.0	(1/1)		100.0	(1/1)
Fungi	82.1	(2886/3515)		94.5	(3322/3515)	86.3	(3351/3881)	85.2	(3046/3576)		94.9	(3427/3612)
Bacteria	<0.1	(75/122777)		<0.1	(9/122777)	<0.1	(9/123545)	<0.1	(336/123872)		<0.1	(5/124626)

GA14F/RM4R[2]		GA18F/RM7R[2]		GA18F/RM8R[2]		GA20F/RM7R[2]		GA20F/RM8R[2]		GA20F/RM9R[2]	
D6		D8		D8		D8		D8-D9		D8-D9	
202 bp		424 bp		572 bp		348 bp		406 bp		505 bp	
95.5	(592/620)	50.1	(327/653)	96.0	(620/646)	97.7	(638/653)	97.8	(632/646)	98.0	(632/645)
96.7	(526/544)	86.3	(465/539)	89.2	(472/529)	91.7	(494/539)	64.1	(50/78)	96.6	(510/528)
35.7	(20/56)	96.4	(54/56)	100.0	(56/56)	96.4	(54/56)	100.0	(56/56)	100.0	(561/561)
91.9	(68/74)	97.1	(68/70)	95.5	(64/67)	98.6	(69/70)	97.0	(65/67)	98.5	(65/66)
100.0	(1/1)	100.0	(1/1)	100.0	(1/1)	100.0	(1/1)	100.0	(1/1)	100.0	(1/1)
98.1	(104/106)	61.1	(69/113)	61.6	(69/112)	95.6	(108/113)	97.3	(109/112)	98.2	(110/112)
80.0	(4/5)	0.0	(0/5)	0.0	(0/5)	100.0	(5/5)	100.0	(5/5)	100.0	(5/5)
100.0	(35/35)	48.6	(17/35)	45.7	(16/35)	77.1	(27/35)	74.3	(26/35)	77.1	(27/35)
45.0	(9/20)	0.0	(0/20)	36.8	(7/19)	0.0	(0/20)	100.0	(19/19)	100.0	(19/19)
100.0	(9/9)	0.0	(0/9)	0.0	(0/9)	0.0	(0/9)	0.0	(0/9)	0.0	(0/9)
100.0	(1/1)	100.0	(1/1)	100.0	(1/1)	100.0	(1/1)	100.0	(1/1)	100.0	(1/1)
95.8	(3480/3631)	90.7	(2943/3244)	88.9	(2849/3205)	95.4	(3096/3244)	94.0	(3096/3292)	94.4	(3027/3205)
<0.1	(7/124788)	<0.1	(28/124775)	<0.1	(10/124065)	<0.1	(49/124775)	<0.1	(10/124065)	<0.1	(27/124041)

Primer sets were tested for matches to sequences in the SILVA database (v.132) using TestPrime with the parameters (maximum number of mismatches of five bases and length of 0-mismatch at the 3′ end of three bases). TestPrime computes coverages for each taxonomic group by running *in silico* PCR on the SILVA database via sorting database sequences into “match”, “mismatch” and “nodata (sequences not covering the primer match position)”. The frequencies of “match” sequences among “match” and “mismatch” sequences are shown as percentages with the sequence numbers in parentheses.

[1] Only 17 bases from the 3’ was used for the primer EukBr because many sequences in the SILVA database lacks the corresponding 5’ region.

[2] Target variable regions and amplicon sizes based on the *S. cerevisiae* rRNA gene (NC_001144) are shown below the primer names.

[3] These primer sets could not be evaluated by the TestPrime.

Table S4. Summary of numbers of sequence reads and detected OTUs.

		EMP (1391F/EukBR)	563F/1132R	616F1132R	1183F/1631R	DM568F/RM2R	RM2F/RM3R	GA12F/RM4R	GA20F/RM9R
WR4	Num of Reads[1]	13025 (17667)	4285 (25474)	15753 (16307)	18996 (18996)	48866 (48866)	27792 (27792)	12072 (12072)	13899 (13899)
	Num of OTUs[1]	117 (259)	129 (1516)	414 (500)	289 (289)	385 (385)	155 (155)	187 (187)	250 (250)
WR5	Num of Reads[1]	21053 (23017)	9574 (27739)	19317 (19375)	19293 (19293)	43225 (43225)	27554 (27554)	10823 (10823)	17161 (17163)
	Num of OTUs[1]	82 (166)	161 (1264)	354 (375)	168 (168)	169 (169)	56 (56)	141 (141)	179 (181)
WR6	Num of Reads[1]	17304 (24203)	7750 (29832)	20698 (20992)	23037 (23037)	44286 (44286)	31753 (31753)	12996 (12996)	22461 (22463)
	Num of OTUs[1]	103 (217)	258 (1235)	493 (528)	322 (322)	198 (198)	148 (148)	227 (227)	299 (301)
WR7	Num of Reads[1]	13734 (24795)	3846 (31222)	16914 (17969)	17365 (17365)	42145 (42145)	30170 (30170)	12128 (12128)	14914 (14914)
	Num of OTUs[1]	136 (313)	151 (1524)	394 (469)	358 (358)	232 (232)	131 (131)	204 (204)	218 (218)
WR8	Num of Reads[1]	15104 (20068)	6480 (31761)	19130 (19437)	22575 (22575)	52606 (52606)	29309 (29309)	15091 (15091)	16324 (16326)
	Num of OTUs[1]	101 (229)	94 (1243)	319 (358)	174 (174)	177 (177)	62 (62)	158 (158)	151 (153)
ZR4	Num of Reads[1]	9963 (21356)	2820 (30190)	13878 (14949)	20592 (20592)	33541 (33541)	25924 (25924)	9196 (9196)	17680 (17684)
	Num of OTUs[1]	121 (201)	102 (1647)	209 (303)	339 (339)	108 (108)	44 (44)	256 (256)	183 (187)
MB1	Num of Reads[1]	3465 (12234)	499 (28553)	5468 (6146)	16258 (16259)	37951 (38009)	35874 (35879)	5095 (5095)	19028 (19476)
	Num of OTUs[1]	224 (376)	68 (1645)	281 (363)	908 (909)	407 (413)	253 (254)	156 (156)	467 (486)

[1] Eukaryotic read and OTU numbers are shown. Total number of reads/OTUs are shown in parenthesis.

In a separate excel file.

Table S5. Taxonomic classification of eukaryotic reads of all faecal samples and primer sets at the SILVA Level 1 (Dataset 1)

Table S6. Taxonomic classification of eukaryotic reads of all faecal samples and primer sets at the SILVA Level 4 (Dataset 1)

Table S7. Taxonomic classification of eukaryotic reads of all faecal samples and primer sets at the SILVA Level 7 (Dataset 1)

Table S8. Taxonomic classification of eukaryotic reads of all faecal samples and primer sets at the SILVA Level 10 (Dataset 1)

Table S9. Taxonomic classification of eukaryotic reads of all faecal samples and primer sets at the SILVA Level 1 (Dataset 2)

Table S10. Taxonomic classification of eukaryotic reads of all faecal samples and primer sets at the SILVA Level 4 (Dataset 2)

Table S11. Taxonomic classification of eukaryotic reads of all faecal samples and primer sets at the SILVA Level 7 (Dataset 2)

Table S12. Taxonomic classification of eukaryotic reads of all faecal samples and primer sets at the SILVA Level 10 (Dataset 2)

Table S14. Evaluation of the taxon-specific primer sets and their coverages in 14 taxonomic groups by TestPrime (maximum number of mismatches of four bases and length of 0-mismatch at the 3’ end of three bases)

Table S13. Evaluation of A) 18S and B) 28S primer sets whose amplicon size is out of the target range for matches to the sequences of 13 taxonomic groups.

A)

Taxonomy	Representative species	566F/1200R[1] V4-V5 634 bp	
Nematoda	Roundworm, Filaria	94.6	(2043/2159)
Platyhelminthes	Tapeworm, Fluke	86.4	(1672/1953)
Acanthocephala	Spiny-headed worm	85.7	(54/63)
Coccidia	Coccidium	77.4	(65/84)
Cryptosporida	Cryptosporidium	94.5	(52/55)
Haemosporidia	Plasmodium	95.9	(142/148)
Fornicata	Giardia	76.2	(16/21)
Discicristata	Trypanosoma, Leishmania	45.3	(384/847)
Parabasalia	Trichomonas	82.2	(249/303)
Entamoebida	Entamoeba	100.0	(4/4)
Longamoebia	Acanthamoeba	89.8	(115/128)
Fungi		95.2	(14289/15003)
Bacteria		<0.1	(12/575261)
Euteleostomi	Mammal	72.7	(784/1079)

B)

Taxonomy	Representative species	RM7F/RM9R[2] D9 180 bp	
Nematoda	Roundworm, Filaria	96.7	(624/645)
Platyhelminthes	Tapeworm, Fluke	93.2	(492/528)
Acanthocephala	Spiny-headed worm	94.6	(53/56)
Coccidia	Coccidium	77.3	(51/66)
Cryptosporida	Cryptosporidium	100.0	(1/1)
Haemosporidia	Plasmodium	92.0	(103/112)
Fornicata	Giardia	100.0	(5/5)
Discicristata	Trypanosoma, Leishmania	100.0	(35/35)
Parabasalia	Trichomonas	94.7	(18/19)
Entamoebida	Entamoeba	100.0	(9/9)
Longamoebia	Acanthamoeba	100.0	(1/1)
Fungi		91.8	(2946/3209)
Bacteria		<0.1	(11/124041)
Euteleostomi	Mammal	83.3	(334/401)

Primer sets were tested for matches to sequences in the SILVA database (v.132) using TestPrime under parameters (maximum number of mismatches of 4 bases, and length of 0-mismatch at 3' end of 3 bases). TestPrime computes coverages for each taxonomic group by running an in silico PCR on the SILVA database by sorting database sequences into "match", "mismatch" and "nodata (sequences not covering the primer match position)", The frequencies of "match" sequences out of "match" or "mismatch" sequences are shown in percentages.

[1] Target variable regions and amplicon sizes based on *S. cerevisiae* rRNA gene (NC_001144) were shown below primer names.

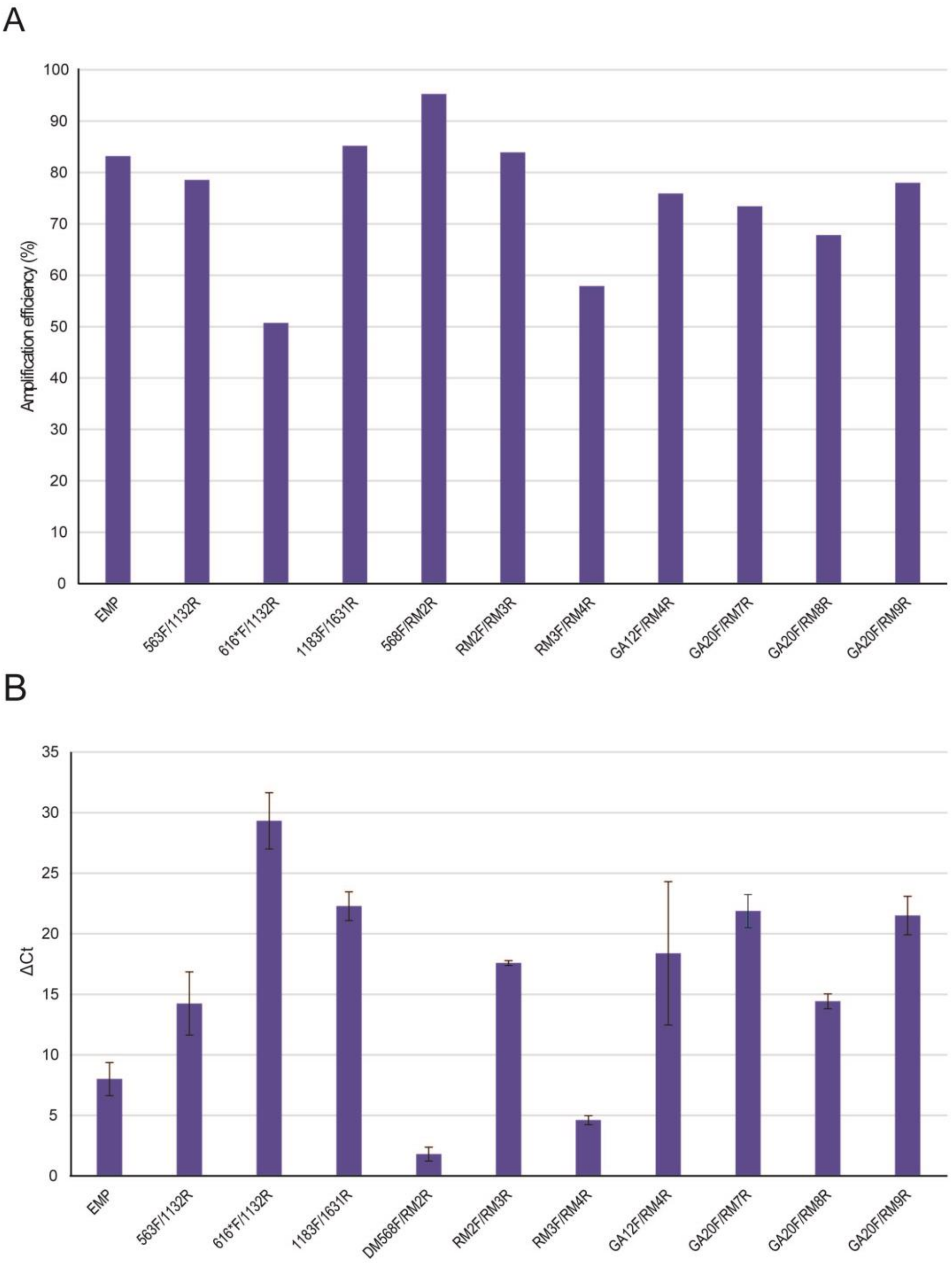

Fig. S1. PCR amplification efficiency and bacterial amplification of 18S and 28S primer sets. (A) PCR efficiencies were calculated for each primer set targeting 18S or 28S rRNA genes using *C. elegans* genomic DNA as templates. (B) Differences of Ct (Cycle threshold) values between the two templates (Ct with 0.1 ng bacterial DNA minus Ct with 0.1 ng *C. elegans* genomic DNA) were shown for each primer set. Error bars represent S.E. from triplicate data.
